# Breaking the taboo: eight Swedish clinical psychologists’ experiences of compassion fatigue

**DOI:** 10.1080/17482631.2020.1785610

**Published:** 2020-07-07

**Authors:** Malin Norrman Harling, Elisabeth Högman, Elinor Schad

**Affiliations:** Department of Psychology, Lund University, Lund, Sweden

**Keywords:** Clinical psychologists, compassion fatigue, organizational factors, personal strategies, semi structured interviews, Sweden, thematic analysis

## Abstract

**Purpose:**

The aim of the study was to investigate the participating psychologists’ experiences with compassion fatigue, and to identify individual, interpersonal, and organizational factors and strategies perceived as contributing or protecting in relation to compassion fatigue.

**Methods:**

Semi-structured telephone interviews were conducted with eight psychologists (three men and five women) with more than five years of experience in clinical practice. The interviews were analyzed with thematic analysis, generating five themes.

**Results:**

These were organizational and task specific factors which the participants felt contributed to their compassion fatigue (“mission impossible” and “emotional strain”), experiences of compassion fatigue (“consequences”), interpersonal factors that were perceived as contributing or protecting (“interpersonal factors”), and individual factors that were perceived as protecting (“shielding and strengthening factors”).

**Conclusions:**

It was found that all of the participants had experienced negative impact of compassion fatigue on their personal lives. A large quantity of patients, and complex patient cases, as well as high expectations on the psychologists were experienced as contributing factors. Collegial support, an empathetic boss, a high degree of agency at work, and fulfiling activities outside of work were experienced as protecting against compassion fatigue.

## Introduction

The ideal psychologist is often seen as a person with unwavering empathy, whose main goal in life is to help others. This expectation on psychologists, often by psychologists themselves, leads to potentially detrimental consequences, where the individual forsakes reflection and recuperation. A question of sustainability for the psychologist profession can thus be raised. Which factors are important if psychologists are to maintain a professional well-being throughout their careers?

The number of sick days per year for psychologists are among the highest for professionals with higher education in Sweden (Försäkringskassan, 2018). This rising number can have many explanations, but as Westberg (2018) argues, the main reason is organizational factors that limit psychologists’ professional freedom, and cause ethical stress. According to Westberg, these detrimental organizational factors also contribute to compassion fatigue, a symptom of stress, which can lead to long-term sick leave if left untreated. Long-term sick leave is costly for the employee, as well as for the employer, and sick-presence at work while suffering from compassion fatigue has been shown to negatively affect clients, families, friends, and colleagues (B. Bride et al., 2007; Negash & Sahin, 2011). In addition to the financial and social costs of compassion fatigue, a high employee turnover rate and frequent vacancies have also been identified as risks to patients (Inspektion för vård och omsorg, 2017). In this study, the primary focus is on psychologists’ experiences of compassion fatigue and not, although interesting, the broader effects on other groups.

### Definition

According to Figley and Ludick, the term *compassion* can be defined as “ … a deep awareness of the suffering of another coupled with the wish to relieve it. It is a kind of focused, action-oriented empathy … ” (Figley & Ludick, 2017, p. 574). Compassion is therefore an essential tool and motivation for the professional helper. Compassion contributes to satisfactory patient care, and professional well-being, which incentivizes research on the subject of compassion fatigue.

Compassion fatigue refers to the negative effects of being exposed to patients’ suffering (Bride et al., 2007). It is not recognized as a psychiatric diagnosis in DSM-V or ICD-10 as of 2019, but has been studied since the 1990’s. The term *compassion fatigue* was first mentioned in an article by Carla Joinson in 1992. She interviewed an experienced crisis counsellor, who differentiated between burnout among caregivers, and burnout among other professions. Joinson argued that nurses often considered themselves caregivers or nurturers, and as such were more affected by their patients’ suffering, and more likely to sacrifice their own well-being in order to help others. Joinson titled her article with the question “Burned out and burned up—has caring for others made you too tired to care for yourself?” (Joinson, 1992, p. 116). This question mirrors the continuing research on compassion fatigue, which mainly centers around a lack of self-care and work-life balance.

The symptoms of compassion fatigue have been revised as the understanding of the condition has grown. During the early years of compassion fatigue research, symptoms focused on the effects of working with traumatized patients, such as secondary traumatic stress (Stamm & Figley, 1996). With time, as compassion fatigue has been recognized in other populations, the list of symptoms has broadened. Symptoms include sleep difficulties, emotional exhaustion or withdrawal, difficulties in executive functions, and poor work performance (Bride et al., 2007; Sorenson et al., 2016).

In order to prevent long-term sick leave due to misdiagnosis and erroneous treatment, it is important to recognize compassion fatigue, and differentiate it from other stress related issues. The symptoms of compassion fatigue are screened for with various instruments, such as CFST (Compassion Fatigue Self Test) (Stamm & Figley, 1996), Professional Quality of Life Scale (Pro-QOL) (Stamm, 2010), which is a revision of the CFST, and comprises the three subscales *burnout, compassion satisfaction*, and *compassion fatigue*, and Secondary Traumatic Stress Scale (STSS) (Bride et al., 2004). These different tests screen for various areas of compassion fatigue, and Bride et al. (2007) recommend using broad testing, with multiple tests, as compassion fatigue can affect various aspects of the sufferer’s life.

In similarity with compassion fatigue, *compassion satisfaction* is a state which individuals in helping professions may experience in relation to their work. The state is characterized by feeling satisfied when helping others, and experiencing excitement from work (Stamm, 2010). These feelings are often accompanied by positive thoughts about work, a will to continue working, and a belief that one can make a positive difference for others. While the concept of compassion satisfaction in a lot of ways differs from, and is the opposite of, compassion fatigue, it is possible for an individual to simultaneously experience both states in relation to their work (Stamm, 2010). However, compassion fatigue reduces the individual’s ability to help others, which is thought to lead to reduced compassion satisfaction (Bride et al., 2007).

An overlapping term which is often used in conjunction with compassion fatigue is *secondary traumatic stress*. The terms are sometimes used synonymously as both stem from witnessing the suffering of others. However, compassion fatigue arises from feeling unable to alleviate suffering, while secondary traumatic stress is caused by being exposed to trauma, such as hearing someone else’s traumatic experiences. Such second hand experiences may cause symptoms associated with PTSD, e.g., intrusive thoughts, or hyperarousal (Diehm et al., 2019). In recent research, it has been suggested that compassion fatigue, secondary traumatic stress, and *vicarious traumatization* can be grouped under the term *empathy-based stress* (Rauvola et al., 2019).

The early research on compassion fatigue focused heavily on clinical social workers working with traumatized populations, and the term was used interchangeably with *secondary traumatic stress* (Bride et al., 2007). As research broadened, a clearer distinction was made between the terms, and compassion fatigue was recognized in professions outside the field of trauma treatment. It has now been shown that compassion fatigue occurs in other helping professions as well, such as among special education teachers (Sharp Donahoo et al., 2018), and social workers (Denne et al., 2019).

A common misconception is that the terms *compassion fatigue* and *burnout* can be used synonymously. To clarify, compassion fatigue differs from burnout in rapidity of onset, long-term effects, and severity in symptoms. Compassion fatigue has a quicker onset than burnout, and affects fewer aspects of the sufferer’s life. Burnout causes mental and physical impairments, may require a change of work or career, and can have lifelong detrimental effects. Compassion fatigue, on the other hand, is highly treatable, once identified, and is seldom chronic (Figley, 2002). However, Sorenson et al. (2016) found a positive correlation between compassion fatigue and burnout, suggesting a relationship between the two terms.

### Psychologists in Sweden

To become a licenced psychologist in Sweden, one has to complete a five year long vocational program, work as a psychologist under supervision for one year, and apply for a licence with the National Board of Health and Welfare. Psychologist is a protected title, meaning that only those who have completed, or are undergoing, their supervised year may title themselves psychologists (Psykologförbundet, 2018). In the official records, 70% of Swedish psychologists are identified as female and 30% as male (Socialstyrelsen, 2019).

The availability of psychological help is fairly high in Sweden, compared to other countries. In 2017, there were 8 519 psychologists working in the mental health sector in Sweden, equating to 84 per 100 000 inhabitants (Socialstyrelsen, 2019). This can be compared to Germany, with 49,6 psychologists per 100 000, Finland with 109,5, and the USA with 29,9. Note that these numbers refer to psychologists and do not include psychotherapists with a medical background or other mental health professionals. In Sweden, mental health services are covered by publicly funded insurance (World Health Organization, 2017), meaning in theory that mental health services are available to all citizens.

### Previous research

#### Predicting factors

**Figure 1.**
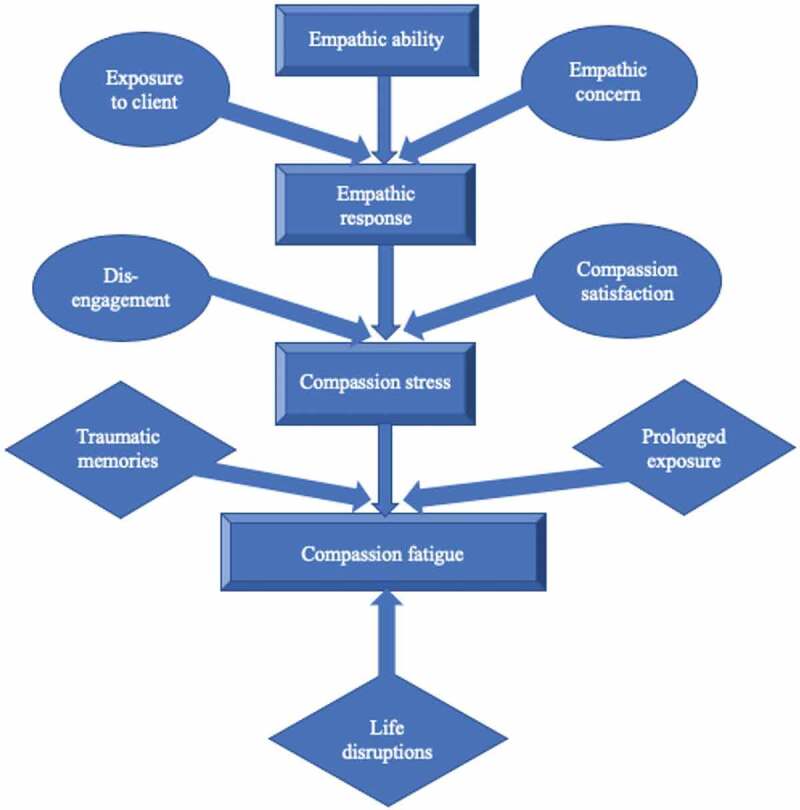
Compassion stress and fatigue model. Adapted from Figley (2002).

Figley (2002) found eight predicting factors for the development of compassion fatigue: empathic ability, empathic concern, exposure to the client, empathic response, compassion stress, prolonged exposure to others’ suffering, traumatic recollections, and life disruptions (see Figure 1). Empathic ability refers to the ability to feel empathy—a cornerstone in any caregiving profession. Empathic concern is the motivation that drives a person to act on their empathy. Exposure to clients refers to the work that involves the client and the professional. When professionals are empathetically engaged in their clients and are unable to help them, they suffer compassion stress (Figley, 2002). This can be compared to *ethical stress*, which arises when the professional is not given sufficient resources to adhere to their professional assessment of the ethical course of action (Forster, 2009). If professionals are subjected to prolonged exposure to the suffering of others, and if they experience traumatic recollections of their own lived experience, or recollections of clients’ trauma, their ability to stave off compassion fatigue is further reduced. These traumatic recollections can be brought on by a certain type of client, whose stories or behaviours trigger negative emotions for the professional. Finally, life disruption, such as unexpected changes in the professional’s family situation, work schedule or health, can further increase the risk of developing compassion fatigue (Figley, 2002).

Negash and Sahin (2011) found a greater risk of compassion fatigue among family therapists working with clients suffering from chronic illnesses or severe depression. Working with victims or perpetrators of child abuse was also identified as a risk factor. Those therapists who had unprocessed difficult family situations of their own were at an even greater risk. However, whether or not a professional’s own experiences of maltreatment affects the risk of developing compassion fatigue has been debated (Craig & Sprang, 2010).

Furthermore, it has been found that organizational factors can predict compassion fatigue. A study on nurses showed a significant correlation between a low level of manager support and compassion fatigue (Hunsaker et al., 2015). This finding was supported by Sorenson et al. (2016), who found certain organizational factors to be contributing to compassion fatigue in nurses: poor work support, shortage of staff, and overall work stress. Craig and Sprang (2010) found a negative correlation between compassion fatigue and evidence-based practices, suggesting that clear guidelines on how to conduct one’s work might protect against compassion fatigue. The fact that organizational factors contribute to compassion fatigue is an important finding that warrants further investigation.

#### Protecting factors

Previous research has indicated that there are organizational factors that protect health care professionals against compassion fatigue, such as professional development opportunities, debriefing, and being mentored (Craig & Sprang, 2010; Sorenson et al., 2016). Having access to personal therapy has also been suggested to decrease the risk of developing compassion fatigue and other work-related stress (Linley & Joseph, 2007) Further research on the protecting effects of organizational factors is required to bring more insight on how to prevent compassion fatigue.

Figley (2002) found that mental health professionals who feel satisfied with their work, and are able to disengage from the caregiving role, experience these factors as protecting against compassion fatigue. He also argues that these self-care practices require recognition and a conscious effort from the professional, and should not be taken for granted.

It has also been shown that personal and individual factors, such as self-care, social support, and optimism increase the resilience against compassion fatigue (Craig & Sprang, 2010; Gentry, 2018). In an interview study on physicians working in palliative care, some of the main findings were individual strategies to prevent compassion fatigue, such as seeking support from colleagues, and maintaining boundaries between work and spare time (Bessen et al., 2019). The importance of collegial support is supported by a study on nurses, where seeking support in colleagues was considered to be a protecting strategy (Yoder, 2010).

Furthermore, possible factors of resilience that could prevent or delay the onset of compassion fatigue in psychologists and psychotherapists have been explored to some extent. Miller and Sprang developed a model for how psychotherapists could avoid developing compassion fatigue or secondary traumatization. Mindfulness, managing rumination, avoiding emotional labour, and recuperation were considered to be important strategies (Miller & Sprang, 2017). Compassion satisfaction and overall job-satisfaction has also been found to be a protecting factor against compassion fatigue amongst psychotherapists (Harrison & Westwood, 2009; Michalchuk & Martin, 2018). Dehlin and Lundh (2018) found that a reflective stance, and supervision, contributed to lower rates of compassion fatigue, raising the question of which other factors could be protecting against compassion fatigue in psychologists.

#### Aims of the study

In light of previous research, the aim of the present study is to explore the participating psychologists’ experiences of compassion fatigue, and identify individual, interpersonal, and organizational factors they perceive as protecting or contributing. A secondary aim is to identify successful coping strategies used by the participating psychologists.

## Method

### Research design

A phenomenological qualitative design was chosen in order to gain insight into the participants’ personal experiences of compassion fatigue in their work as clinical psychologists. Individual, semi-structured telephone interviews were conducted with the participating psychologists. This method was chosen in order to allow participant-generated meanings to come forth through the use of open-ended questions, and a flexible use of the interview template. The research method was based on Willig’s (2013) interpretation of qualitative and phenomenological research, which is in part based on the Husserlian philosophy.

In accordance with phenomenological qualitative design, the aim of this study was to reach each participant’s subjective experience, not an objective truth. In order to do so, preconceived notions must be carefully examined and lain aside. This is referred to as *epoché*, in accordance with the Husserlian philosophy, where one brackets one’s instinctive attitude to allow a new way of viewing the studied phenomenon. Approaching the data in such a way allowed the intentionality of the participant to come forth, rather than the causality of the phenomenon, which is better studied through other methods (Englander, 2016).

The epistemological approach to this study was essentialist/realist and semantic, as the aim of the study was to account for the participants’ experiences as described by themselves, rather than attempting to interpret meaning beyond their words (Braun & Clarke, 2006).

The analysis was carried out with a deductive/theoretical approach (Braun & Clarke, 2006), meaning that the study was based on previous research and understanding of compassion fatigue. In other words, our understanding of the phenomena was based on the notion that compassion fatigue is a work-related state, in accordance with previous research. Furthermore, previous research has found individual strategies to be useful against compassion fatigue, another notion that this study was based on. The aim and research questions of the study were also predetermined, meaning that the research question was consistent and did not evolve through the coding process. Thus, the aim and the interview template used in this study were based on this theoretical framework surrounding the phenomena, and the study was therefore deductive in nature.

Prospective and retrospective reflexivity (Willig, 2013) was used throughout the research process, by recognizing the impact of the researchers’ preconceived notions on the research process, as well as the study’s impact on the researchers.

### Participants and study setting

The participants were recruited through two channels: a social media platform and the email list of a Swedish association for clinical psychologists. The sample was purposively chosen to consist of psychologists who had experiences of compassion fatigue. During August and September 2019, 11 psychologists volunteered for the interviews. Of these, nine sent in the consent form within the set time, and eight were available for interviews during the given time frame. The eight participating psychologists (three men and five women) all had more than five years of experience of clinical practice in Sweden (see Table I), the number of years in the profession ranging from 11 to 16 years (*M* = 13,25, *SD = *1.92). The participants came from publicly funded organizations and worked in general and specialized care. Ages ranged from 38 to 46 years (*M* = 42,75, *SD = *2.38). To ensure the participants’ anonymity, age is not presented in the table. The interviews were conducted by telephone and ranged in duration between 40 and 90 minutes (*M* = 64 min, *SD *= 14 min).

**Table I.** Participants.

Alias	Workplace	Years of experience	Interview duration
Annie	Children. Public sector.	16	70 min
Beatrice	Psychiatry/primary health care. Public sector	15	55 min
Cilla	Social service. Public sector	14	40 min
David	Chronically ill adults and children. Public sector.	11	90 min
Ester	Children. Public sector.	11	58 min
Fabian	Occupational health. Private sector.	13	72 min
Gabriel	Institutional care. Public sector.	12	57 min
Hanna	Primary health care. Public sector	15	69 min

### Data collection

The interview guide (Appendix) included open-ended questions which covered four different topics: 1) the participants’ own experiences of compassion fatigue, 2) strategies for coping with compassion fatigue, 3) which factors in their working environment they considered to be helpful or hindering in relation to compassion fatigue, and 4) how they experienced the impact of compassion fatigue in their personal and working lives. These four topics were chosen after reviewing the literature on predicting and protecting factors, drawing inspiration mainly from Figley’s research (2002).

Two pilot interviews were conducted during the spring of 2019 in order to allow for appropriate revisions to the interview guide, before the study was submitted to the Swedish Ethical Review Authority for approval. Once approved, the interviews were conducted, recorded, and transcribed using equipment without access to an internet connection to ensure data protection. The quotes that are presented in findings were translated from Swedish to English, and the participants’ names have been altered to protect their anonymity.

### Data analysis

The steps of data analysis followed Braun and Clarke's (2006) recommendations for thematic analysis. Firstly, the interviews were transcribed, followed by a thorough familiarization with the data by both researchers separately. While reading through the interviews, initial codes were generated, which were then compared, discussed, and clustered into preliminary themes. These themes were then revised by a second coding of all interviews, and the five main themes were decided on. In order to ensure a proper analysis of the data, the coding steps were carried out by both researchers separately, and were then compared. During the process, the themes were constantly revisited, and a few sub-themes were revised.

### Ethical considerations

The study was approved by the Swedish Ethical Review Authority (application number 2019-02955), provided that the participants’ personal information would be handled in accordance with the EU law of General Data Protection Regulation. Any potential risks of psychological discomfort to the participants were determined to be quite small, as the participants were preumed to be used to introspection through their work as psychologists, and the participants were not pressured on sensitive questions during the interviews. The participants received written and oral information about the study beforehand, and signed a consent form before being interviewed. This form stated the aim of the study, the method of data collection and handling, that participation was anonymous and voluntary, and that participants could withdraw their consent at any time.

## Findings

A thematic analysis of the data was conducted, and five main themes with sub-themes were found; “mission impossible”, “emotional strain”, “consequences”, “interpersonal factors”, and “shielding and protecting factors”. The themes and sub-themes are presented in Table II and are illustrated by shorter participant quotes.

**Table II.** Themes and sub-themes.

Mission impossible			
	Logistical tangle		“I often have to contact different types of aid managers and assisting authorities, like social services and such. There is a lot of additional tasks surrounding what I do.”—David
	Quantity of patients, and high demands		”I definitely think that this huge influx of patients that we have in primary care, that it makes you … that it plays a part in the risk of becoming indifferent.”—Hanna
	Unsustainable framework		“I think it has to do with our participation in decision-making regarding our profession and how we practice, but these decisions are often made by politicians.”—Beatrice
Emotional strain			
	Jesus complex		”(…) you can’t forget that you are a human being just like your patients, with fundamental needs that have to be fulfilled.”—Beatrice
	Forcing compassion		”(…) respect towards the patient that I had in front of me. That it was my duty to be interested, and to focus on that.”—Fabian
	Ethical stress		”We get to hear a lot, we observe huge needs, but we have very little capacity to help.”—Ester
	Difficult fates		“You get to hear a lot of things that affect your view of humanity. And not for the better.”—Fabian
Consequences			
	Highway to burnout		“It’s connected to being burnt out, and not having any more energy. Compassion fatigue is a symptom of that”—Hanna
	Annoyance and categorical thinking		“I just can’t stand that commonplace bickering and whining”—Ester
	Numbness and isolation		“I don’t have the energy to sit there and feel”—Cilla
Interpersonal factors			
	Collegial support		“We worked together as a team. There was always someone who knew sort of what I was doing, I was never alone in it.”—Annie
	Good/bad boss		“My first boss was a disaster. (…) She’s the reason I quit. She made every mistake in the book, which was a disaster for me personally.”—Gabriel
	Friends and family		“I have a stable family situation now, with a long-term partner and children. (…) My partner and I can vent to each other about work, and that’s good”—David
Shielding and Strengthening Factors			
	Controlling circumstances		“It feels like the right thing to do, ethically, the humane thing to do. But if you look at how we are supposed to act, we are supposed to close cases quicker than we oftentimes do”—Cilla
	Professional development		“You need further education and supervision to learn and to develop. This is a strenuous job.”—Annie
	Self-care		“When I notice that I’m starting to lose it, I think “I need to recharge my batteries, but I also have to make sure that I keep my everyday life running”“—Gabriel
	Job satisfaction		“When what I do makes a difference. (…) It’s very satisfying when those hours I’ve spent working with this patient actually makes a tangible difference in that person’s life”—David

### Mission impossible

The participants identified several work-related factors that made their work seem like an impossible mission. A “logistical tangle” was described by several participants, encompassing administrative tasks, and coordinating with other professionals involved with the patients. The participants also mentioned experiencing “quantity of patients, and high demands”, requiring them to treat a large number of patients, and take on complex cases. They also described working within a detrimental and “unsustainable framework”.

#### Logistical tangle

Participants reported time-consuming tasks, such as having to juggle co-workers’ schedules, reaching out to patients’ insurance companies, and documenting every step of the process. One participant described the increased administrative demands at her workplace:

We constantly have to select options, make a bunch of tiny decisions related to statistics, and show that we’re following the guarantee of care [regulated in Swedish law]. We spend so much time proving to the politicians that we’re using our time the way the politicians want us to use our time. It’s incredibly frustrating.—Hanna

Participants expressed that the excessive amounts of documentation around patients forced them to prioritize administration over clinical work. Having to meet the administrative demands, while juggling the logistics of their patients’ cases, was cited as a contributing factor to work-related stress and compassion fatigue.

#### Quantity of patients, and high demands

The sheer number of patients was often mentioned as a stressor, whether it was many patient meetings per day, or a long patient wait-list. One participant described her experiences at a previous workplace:

The demands on quantity were very high. Also, when I worked in psychiatric care, I had a diffuse role as contact person, which had nothing to do with my profession. By the time I quit that job I think I had at least seventy patients with severe mental illness where I was their only contact (…) and that is something that needs to be discussed—is this reasonable?—Beatrice

Several participants also mentioned an increased complexity in the problems the patients presented, compared to when they started working as psychologists. Despite having many years of experience within his field, one participant expressed feeling strained by complex cases:

(…) difficult cases, that really demand a lot of your ability—it can be things that are difficult to get somewhere with, and help the patient as much as you want, and you feel insufficient.—Fabian

These complex problems demanded more resources than the participants were used to, in terms of time, and emotional and mental energy. Some of the participants associated these high demands with an increased risk of compassion fatigue.

#### Unsustainable framework

When discussing their workplace, several participants expressed feeling dissatisfied with the surrounding framework and policies, which were either too narrow, ill-suited to the participants’ preferences, or were not clearly defined. One participant described a mis-match between himself and the frames within which he was required to work:

I like working with changes, not so much with assessments. So it’s not sustainable for me, because I think assessments are really boring to do. I have colleagues who have been here for many, many years, and who think it’s amazing. So it has got more to do with how you are as a person.—Gabriel

This participant said that the framework within which he worked was not sustainable for him, as he had other preferences in work tasks. The discomfort of this mis-match was in part alleviated by his colleagues, with whom he enjoyed working, but he stated that the mis-match had made him consider switching jobs. Another participant who felt more in charge of her framework described that she felt free to plan her work according to her own needs and preferences:

Where I am now, I have a better overview. I book my own appointments with my patients, so I can make sure it’s reasonable.—Beatrice

The ability to book appointments herself helped her manage and handle her workload, as she could plan ahead, and maintain her work-life boundaries. This, she stated, was a protecting factor against compassion fatigue and burnout. In contrast, several participants felt dissatisfied with the lacking regulation and policies at the workplace, which resulted in them having to create a framework for themselves. One participant in particular expressed that the framework at his workplace was too lax:

Sometimes it’s hard to get clear information on how we’re supposed to prioritize, and which target groups and patients I should take in as a psychologist. Like, how I should use my time at work.—David

For this participant, the freedom he experienced in creating his own framework was cited as negative, as it meant taking on managerial responsibilities without having the time or knowledge to do so. Other participants also conveyed a wish for clear leadership, in order to create a sustainable framework, while still being allowed to make independent decisions regarding their practice. Some participants mentioned sustainable framework as protecting against compassion fatigue.

### Emotional strain

The participants described experiencing a “Jesus complex” as they were expected to always show compassion towards others. Some of the participants described a reduced ability to experience and show compassion, and “forcing compassion” as a result. The emotionally strenuous aspects of their work included “ethical stress”, and working with their patients’ “difficult fates”.

#### Jesus complex

Several participants expressed that they felt like it was expected of them to always have the time and energy to help others, always show compassion towards others, and not being negatively affected by listening to tragic and sometimes horrifying details of patients’ lives. One participant described his frustration about the impossible role that he felt obligated to fill as a psychologist:

It’s my experience that people in general think that we’re some sort of freaking superhumans. We’re not supposed to feel, and we’re supposed to hear about all this suffering without being affected by it (…) But we’re human! That’s it. We are humans too. And we have the same needs as everyone else.—Gabriel

Several other participants mentioned meeting similar attitudes, especially in connection to compassion fatigue and its taboo amongst psychologists. Compassion fatigue was thought to be especially detrimental to psychologists, as compassion is viewed as an important working tool, and the lack thereof would impair the psychologists’ work.

#### Forcing compassion

As a compensatory strategy for compassion fatigue, several of the participants conveyed that they had tried to actively force themselves to be compassionate in their work, and personal life. One participant described how she, in her personal life, sometimes pretended to be more engaged than she really was, to hide the fact that she was actually annoyed with the person talking:

It’s like what I said before, it’s like I’m sighing or rolling my eyes on the inside, but I don’t show it. Maybe I become even more engaged to hide the fact that I just don’t have the energy, you know?—Cilla

Several of the participants described similar strategies, mostly in situations with patients where it was crucial for the treatment that they show compassion and emotional presence. One participant described how he would use certain strategies in order to feel and show compassion towards a patient in a situation where it did not come automatically:

The first thing is to remind oneself over and over that all suffering is subjective, and the fact that I’ve met people who’ve had it worse doesn’t mean that the person in front of me isn’t in great need of help and someone to listen to them (…) It’s like, it doesn’t have anything to do with their experience, it’s just my experience as a caregiver who has met these other patients. Trying to remind myself of that is a strategy to keep my ability of compassion intact.—David

Being able to show compassion was described as being one of the most important working tools for psychologists, incentivizing them to force the ability in some situations. Having to force compassion became emotionally draining for some participants, thus contributing to compassion fatigue.

#### Ethical stress

Ethical stress was cited to be a source of emotional strain for all participants. They described feelings of frustration and dejection when their ability to help their patients was hindered by organizational factors out of their control, and stated that the large quantity of patients forced them to prioritize. One of the participants described her ethical stress in wanting to give the patients more than what was possible, given the systemic preconditions:

It’s straining when we don’t have the resources, when we know that we could have done something, but the resources aren’t enough. (…) So we don’t feel like we can stand behind what we deliver.—Ester

This sentiment recurred in all of the interviews conducted in this study, where participants described their frustration and anger in knowing what is best for the patients, but not being able to provide it. Several participants identified ethical stress as a direct cause of compassion fatigue.

#### Difficult fates

All participants mentioned being emotionally affected by meeting patients, and getting to share their difficult fates. One participant described what types of patient cases that would affect him the most in his work:

It’s a draining job to meet people who are sick. And one thing that’s draining, that I’d like to add, is when people have progressive illnesses (…). We know that they’ll never get well and that we’ll never be able to offer them any treatment or cure it. That’s really strenuous of course.—David

Exposure to patients’ difficult fates, and how the participants were negatively affected by these, often recurred throughout the interviews. In contrast, some participants stated that listening to their patients’ narratives was part of what made their work meaningful, however hard it was. In summary, the participants conveyed that listening to, and working with, “difficult fates” could be both rewarding and emotionally draining.

### Consequences

The participants’ subjective experiences of compassion fatigue were varied, as were the consequences in their professional and personal lives. The participants described having general symptoms of stress in conjunction with compassion fatigue (“highway to burnout”). “Annoyance and categorical thinking” were identified as experienced symptoms of compassion fatigue, as well as “numbness and isolation”.

#### Highway to burnout

When asked about their experience of compassion fatigue, most participants mentioned general symptoms of stress, such as physical fatigue, cynicism, rumination and signs of burnout. One participant described her experiences of burnout symptoms in conjunction with compassion fatigue:

I’ve had these setbacks with burnout … I was sitting in my car after an intense week, where I’ve done lots of travel and done things in other places, in my job. And afterwards, I had to stop the car and sort of let it spin for a while, and get out. And I got so scared, I thought “Dammit! My brain is whacked now.”—Hanna

Similar descriptions of stress in general recurred throughout the data, where participants associated compassion fatigue with burnout, and other stress symptoms.

#### Annoyance and categorical thinking

When asked about their experiences of compassion fatigue many participants described how the state affected their thought patterns. Several participants mentioned that they became annoyed more easily, or were more categorical in their views and assessments. One participant described the annoyance she would experience when having to listen to other people’s problems in her personal life:

In my personal life, I easily feel like people make something out of nothing, I think “God, they whine so much” (…) I’m sick of them whining and just seeing problems.—Cilla

Other participants also mentioned being annoyed with friends and family, but not so much with patients. Nearly all participants stated that their annoyance primarily affected their personal lives. However, some participants admitted that they, or their colleagues, had become categorical in relation to their patients at some point in their careers:

You can tell when a colleague is sick and tired, and you notice that this person is affected, and you can sort of deal with an increased prevalence of categorical thinking. If I get very categorical, my colleagues will notice, and I’ll notice it in myself as well.—Fabian

Some participants conveyed that compassion fatigue caused them to not mentalize properly, resulting in loss of perspective, misunderstandings, and annoyance. The effects of this were first seen in the participants’ personal lives, but spread to their professional lives in time.

#### Numbness and isolation

Several participants conveyed that they sometimes felt numb and emotionally distant in their relationships, lacking the energy to be compassionate and understanding towards others, especially in their personal lives. One participant stated that she sometimes did not have the energy to empathize with others outside of work:

It’s maybe like a stress that builds up, that I don’t want people to come to me and vent too much, in my personal life. (…) But it’s like a stress about finding myself in a situation that I don’t have the energy to be in. I can’t be the one who’s always so understanding, I want to do something else. I’ve been compassionate enough.—Cilla

In addition to emotional numbness, a few participants conveyed that they sometimes needed physical isolation from people in their personal lives. This was described by one participant in the following way: “I’m not fully present at home, with my kids, like that. I have a need to withdraw a little, to be alone”. These types of statements were corroborated by several participants, describing a need to withdraw, physically or emotionally, as a result of compassion fatigue.

### Interpersonal factors

The participants cited interpersonal relationships as being an important factor in relation to compassion fatigue. The experiences of this were grouped into “collegial support”, “good/bad boss”, and “friends and family”. The theme contained negative as well as positive aspects, since interpersonal factors could be both contributing and protecting against compassion fatigue, according to the participants.

#### Collegial support

When facing difficult times at work, support from colleagues was stated as an important protecting factor. One participant stated repeatedly that she stayed at her current workplace because of her colleagues, despite being very strained otherwise:

One thing that makes me want to keep working here is my lovely colleagues. (…) I would not choose to work alone. That would be very lonely. In professions where you have each other, laugh together, that’s a strategy to cope.—Ester

Other participants corroborated this, and said that the informal support they felt, such as during lunch breaks, contributed to a healthy and sustainable work climate. However, not all participants had the same experiences. Some stated that the climate was closed and detrimental to their well-being at work. One participant conveyed that she felt worried about not being able to follow her own moral compass, for fear of jeopardizing the team unity:

It is pretty clear that there are political differences within the team, how things are viewed. And that’s pretty difficult. In some other cases, I have no difficulties taking a stance, but here I feel like I won’t take a stance, because it would make my work situation worse.—Hanna

Similar statements about the negative effects of a closed climate recurred in other interviews. Whether the climate was cited as positive or negative, all participants stated that they felt able to talk to colleagues about difficulties at work. This could however lead to rumination, when the group amplified each other’s complaints. One participant mentioned seeing this often: “You can always ruminate. Ruminating in a group is easily reinforced. (…) It is very rewarding”.

In summary, supportive colleagues were cited as protecting against compassion fatigue, while a closed climate was considered a contributing factor. In addition, some collegial support could become detrimental when turned into rumination.

#### Good/bad boss

The participants stated that their bosses’ personality and leadership style impacted the employees’ well-being at work. One participant described the importance of having a boss who looked out for her, and prioritized her well-being:

She’s that boss who’s like … she brings it up and says “you need more resources now, because this is really hard on you”. It means so much that someone tries … (…) that someone acknowledges those needs.

- Annie

Other participants also cited having caring and supportive bosses. However, not all participants shared this experience. One recurring theme in the data was inadequate leadership—either a passive boss who was dismissive of their employees’ needs, or an active boss whom the participant felt mistreated by:

We had this new boss who was awful, really awful. I believe she had a personality disorder. (…) I never thought that I would be treated that way or see my colleagues be treated that way.—Annie

“Bad bosses” reduced the participants’ feelings of agency, and set unreasonable demands, forcing the participants to prioritize work over recuperation. This was said to decrease the participants’ protection from compassion fatigue.

In summary, having a supportive boss was cited to be a protecting factor against compassion fatigue, while an inadequate boss was considered a contributing factor.

#### Friends and family

Family members, partners, children, and friends were all cited as sources of comfort by the participants. For some, being able to vent to another person about work was enough, and others said that having children helped distract them from thoughts about work, even though parenthood came with its own challenges:

(…) The fact that I have kids. It can be hard. Their needs are so immediately present that you have to acknowledge them. I think that helps you in a way, to let go of work, because this is now.—Annie

This statement was corroborated by other participants, who stated that being a parent helped ground them during difficult times. Other participants stated that personal crises, such as sudden deaths, divorce, or illness, exacerbated their compassion fatigue and overall stress:

In that time, or some time before that, a family member got really ill, which took up a lot of my personal time. That was the tipping point, because it became really hard for me to manage my job.—Beatrice

This experience was corroborated by other participants, who mentioned other negative events to be a contributing factor to their compassion fatigue. Therefore, “friends and family” were cited to be mostly helpful in staving off compassion fatigue, and sometimes exacerbating work-related stress.

### Shielding and strengthening factors

The participants described strategies and factors that they perceived to be protecting against compassion fatigue, and increasing their resilience. They described how they would protect themselves through “controlling circumstances” and practicing “self-care”. They also listed “job satisfaction” and the opportunity to engage in “professional development” as important factors of resilience.

#### Controlling circumstances

Most participants stated that they had taken control of their work environment, in one way or another, in order to increase their well-being at work. They described various strategies to do this, such as setting healthy work-life boundaries, letting go of perfectionism, or switching jobs altogether. One participant found that monitoring his own psychological state was an important strategy that enabled him to make necessary changes to his circumstances:

I’m much better at noticing my compassion fatigue now. I can see what’s happening (…) and I can try and change things. (…) Like “oh, now this happened, I have to change tracks here”—Fabian

Several participants stated that being able to recognize signs of deteriorating well-being was imperative in order to make adequate changes. One participant described how she would actively attempt to lower her expectations of herself, to a more realistic view of what is possible during a session:

(…) being super clever and prepared with every patient—that’s not possible. A lot is routine now. If I look back on a session and think “I should have done this, I could have achieved that boost in the treatment if I had prepared better, but I haven’t”. Then it might be better if the patient gets an extra session, and that’s that.—Hanna

Several other participants also stated that they struggled with perfectionism, and that they had difficulties lowering their expectations on themselves.

Another participant described how he realized through becoming compassion fatigued that he had to switch jobs: “It was terrible to have to change jobs because I wasn’t happy with it, but it was like I realized that I couldn’t do this anymore, thanks to my compassion fatigue. It just wasn’t sustainable.” Similar statements about strategies to control circumstances were made by other participants. Most participants stated that taking control over circumstances in their work life was an important strategy to protect themselves from compassion fatigue.

#### Professional development

All participants conveyed that engaging in professional development was an important factor in their overall well-being at work. Education and supervision were considered to be important elements in professional development. One participant described how she was professionally enriched by being given the opportunity to specialize in clinical psychology, and that this protected her against compassion fatigue:

I think it [education] has contributed to me feeling less compassion fatigued, because I get lots of input from the university, and I get feedback on my work regularly. I get to record on video and I feel like I evolve, and that I get to rehearse my knowledge, and gain new knowledge.—Beatrice

This sentiment was confirmed by several participants, such as one participant who mentioned both education and supervision as important factors in professional development: “You need further education and supervision to learn and to develop. This is a strenuous job.” Overall, professional development, as well as longer experience of working as a psychologist, were considered to be protecting factors against compassion fatigue, as cited by the participants.

#### Self-care

Participants mentioned activities that they like to engage in during their spare time, in order to gain new energy. Some mentioned spending time in nature, being with friends and family, or exercising. Other participants also emphasized the importance of filling basic needs through eating well and getting enough sleep. One participant explained the importance of engaging in fulfiling activities outside of work:

And that you have to take care of yourself during your spare time, to replenish yourself, that you find whatever it is for you or me. It can be different, of course, but you have to learn how to enrich yourself.

- Annie

This statement was corroborated by most participants. Other participants mentioned going to therapy as an essential element in their self-care routine: “(…) go to therapy yourself sometimes, just to keep an eye on your own well-being. I think that’s good”. In summary, finding ways of recuperating in one’s spare time was stated as an important element in coping with a strenuous job, and was considered as protecting against compassion fatigue.

#### Job satisfaction

When asked about what they enjoyed about their work, participants often described feeling compassion satisfaction, in the sense that they experienced joy in being able to help their patients. A few participants described other factors that gave them satisfaction, such as feeling appreciated by co-workers, or enjoying the diversity in their tasks at work. One participant described her experience of compassion satisfaction:

It’s so powerful, getting to be a part of, and hopefully seeing the helping process, it’s so real, it can be life or death. It feels very meaningful, and amazingly alive, and I often describe it as magic.

- Annie

Similar descriptions of compassion satisfaction recurred in other interviews as well, and all participants stated that they experienced job satisfaction to some degree in their current job. The participants described that they had experienced both job satisfaction and compassion fatigue in relation to the same job, and that one did not exclude the other.

## Discussion

### Mission impossible

One of the aims of the study was to identify organizational factors that could be protecting against, or contributing to, compassion fatigue. One of the most commonly recurring factors in this study was time management, and the degree of autonomy the participants experienced in their work.

The participants stated that most of the challenges they faced in their work were manageable, if they were given enough time. However, many of them conveyed that they were unable to complete their tasks during regular working hours, and that they either had to work overtime, or lower the quality of their work. This decreased the participants’ time for recuperation and reflection, and added stress that would otherwise be manageable. It also led to ethical stress, as the participants felt the need to prioritize between their own health and well-being, and the quality of the patients’ care.

The stress of managing a high workload was cited as a cause of compassion fatigue by the participants, supporting previous research (Sorenson et al., 2016). The distinction between work-related stress and compassion fatigue is therefore difficult to make. Several participants conveyed that they often experience the two conditions in conjunction with each other. Therefore, moderating the psychologists’ workload, and thus their work-related stress, might decrease the risk of developing compassion fatigue. However, maintaining the status quo, with extensive overtime and few opportunities to recuperate, has been shown to increase the risk of high turnover rates and long-term sick leave, which in turn is costly to the employer (Westberg, 2018), and lowers the quality of the care available to the patients (Inspektion för vård och omsorg, 2017). This incentivizes finding ways to support health care employees against compassion fatigue, as it lowers the cost for the employers, and improves patient care.

Furthermore, autonomy was perceived as a protecting factor against compassion fatigue. However, a few participants mentioned negative experiences of passive management giving them too much autonomy, forcing them to take on managerial responsibilities, such as prioritizing between patient groups. This uncertainty was said to lead to a higher workload, less job satisfaction, more ethical stress, and compassion fatigue. These statements by the participants further support research by Craig and Sprang (2010), showing that professionals whose work is evidence based suffer less compassion fatigue. Therefore, the effect of perceived control and framework on compassion fatigue is of interest for further research.

### Emotional strain

“Ethical stress” was a prominent sub-theme mentioned by all participants, and is similar to what previous research has identified as *compassion stress*—a state which occurs when the professional is empathetically engaged, but unable to help (Figley, 2002). The participants conveyed that they experienced ethical stress when they were unable to give their patients the help they needed, due to organizational and systemic factors out of their control, such as high demands on patient influx. Sometimes, the experience of ethical stress was intertwined with the emotional strain of listening to “difficult fates”, especially in cases where there was little or nothing that the participants could do to alleviate their patients’ suffering. Previous research has shown that therapists working with clients with chronic illnesses or severe depression were at a higher risk of developing compassion fatigue (Negash & Sahin, 2011), which is supported by the participants’ experiences in this study.

The content of the sub-theme “difficult fates” is similar to what Figley (2002) identified as *traumatic recollections*. However, this term postulates that the prolonged exposure involves trauma, which is not confirmed by the experiences of the participants in this study. It seems like the exposure of suffering could bring psychological discomfort, with or without the aspect of trauma. This exposure is of course an inevitable part of working as a psychologist, which brings up questions of how psychologists’ work environment can be altered in order to alleviate these effects. The findings of this study provide several examples of organizational factors (“good/bad boss”, “professional development”, “job satisfaction”), individual factors (“controlling circumstances”, “self-care”) and interpersonal factors (“collegial support”, “friends and family”) which could make the work situation of psychologist more sustainable, and protect them from developing compassion fatigue.

Furthermore, it was found that several participants identified the role of the psychologist, and the expectations that come with this role, as a risk factor. For the purpose of this study, and with inspiration from one of the participants’ use of the word “Jesus-mentality”, this sub-theme was named “Jesus complex”. A similar phenomenon was described by Joinson (1992).

Several participants of this study mentioned that they felt like it was expected of them to always be compassionate, even in their personal lives. These expectations could be viewed as a contributing factor to compassion fatigue, making psychologists overexert themselves in order to meet such expectations. Furthermore, several participants experienced a taboo surrounding compassion fatigue, because of the “Jesus complex”, making it harder to seek support. Some participants described how they would “force compassion”, in order to compensate for the fact that they suffer from compassion fatigue. It can be speculated that this behaviour could cause more compassion fatigue and emotional strains.

Since the research on “Jesus complex” (or saviour complex) is sparse, further research on the subject is warranted. The term requires operationalization in order to reach a higher understanding of the phenomenon, and further research of the assumable covariation between the complex and compassion fatigue is necessary in order to draw such conclusions.

### Consequences

All participants conveyed that they had experienced compassion fatigue to some degree. Most participants described feeling annoyed or that they engaged in categorical thinking as a result of compassion fatigue. Others described feeling emotionally numb and needing to withdraw from social contexts, a finding supported by previous research (Bride et al., 2007; Sorenson et al., 2016).

Of interest is that several participants conveyed that they primarily experienced these consequences in their personal lives, leaving their professional performance relatively intact. There might be several explanations for this, one of them being the participants’ high expectations of themselves, not allowing their compassion fatigue to interfere with the patient’s treatment. Some of the participants, in addition, stated that their work performance had suffered due to compassion fatigue, a finding that is supported by Bride et al. (2007).

The annoyance described could be interpreted as a healthy reaction to overwhelming expectations, motivating the individual to set appropriate boundaries. Furthermore, the emotional numbness described by the participants may function as a coping mechanism. A few participants described this symptom in terms of habituation, which in some cases would protect them against becoming too emotionally invested in their work. However, a majority of the participants described annoyance and numbness as involuntary, and sometimes detrimental.

Furthermore, it was found that the participants often had experienced other symptoms of stress or burnout in conjunction with compassion fatigue, which is supported by Bride et al. (2007). Previous research has shown a positive correlation between compassion fatigue and burnout (Sorenson et al., 2016). This further supports the assumable connection between burnout and compassion fatigue.

### Interpersonal factors

Interpersonal factors were found to be both contributing and protecting against compassion fatigue. Collegial support was often identified as a protecting factor against compassion fatigue. This supports recent conclusions drawn by Bessen et al. (2019), who argued that health care professionals need built-in time to share patient experiences with their colleagues. Due to the high demands they face at work, our participants stated that they often forsake this sharing of experiences, increasing the risk of compassion fatigue.

Leadership was considered an important factor by all participants. An empathetic boss who provided a clear framework without micromanaging the participants’ quantity of patients or work methods, was considered a protecting factor against compassion fatigue. On the other hand, a non-supportive boss was considered a contributing factor, in line with findings by Hunsaker et al. (2015).

Figley (2002) reported that life disruptions such as divorce, death, or a changed family situation could contribute to compassion fatigue. This was supported by the participants in this study who stated that crises in their personal lives had contributed to their compassion fatigue. Conversely, experiencing stable personal relationships can be viewed as protecting against compassion fatigue. Several participants stated that having small children helped them disengage from work during their spare time, which is supported by research on work-life balance (Bessen et al., 2019; Figley, 2002).

### Shielding and strengthening factors

Several participants mentioned strategies to control their work environment in order to increase their own well-being, such as setting healthy work-life boundaries. Participants also stated that they monitored their psychological and physical well-being through various practices, such as therapy and self-reflection, supporting previous research by Linley and Joseph on the protecting effects of therapy on compassion fatigue (2007). This self-monitoring was integral to the participants' ability to act to improve their well-being, but was cited as being a hard-earned lesson, as many participants described that they had learned to monitor themselves only after reaching a point in their professional lives where they risked developing compassion fatigue or burnout.

Different aspects of professional development were also considered by the participants to be shielding and strengthening factors, which is also supported by previous research (Sorenson et al., 2016). Participants mentioned education, as well as supervision, as protecting factors against compassion fatigue. The protecting aspects of supervision is also found in previous research on psychologists (Dehlin & Lundh, 2018).

All participants conveyed that they experience job satisfaction to some degree in their current job, which previous research has shown to be a protecting factor against compassion fatigue (Figley, 2002). Job satisfaction was often described by the participants in this study in terms of compassion satisfaction. Several participants described experiencing both compassion satisfaction and compassion fatigue in relation to their current jobs, supporting the current understanding of the phenomena (Stamm, 2010).

When asked about what they do to protect themselves from compassion fatigue, most participants mentioned self-care as an important tool. The resilient properties of self-care are supported by previous studies (Craig & Sprang, 2010; Gentry, 2018; Sorenson et al., 2016). Such practices could include being in nature, spending time with friends and family, or exercising. Since the importance of self-care is prominent in this study, we draw the conclusion that further education on the issue is warranted amongst psychologists. For instance, psychologists might be helped by a larger emphasis on the importance of self-care in their training and education.

### Personal reflexivity

In accordance with Willig’s (2013) definition of reflexivity we would like to acknowledge the fact that our personal views and theoretical understanding of the world has shaped our research process, and that the research process in turn, has changed us. Malin Norrman Harlingand Elisabeth Högmanwere Masters Students in the Clinical Psychology Program while working with this study, possibly influenced by an inexperienced and naïve view of the profession. We are all three certainly shaped by our education as psychologists, and the fact that the participants belong to our own in-group. All of us identify as female, and are Swedish nationals of middle class upbringing, and are therefore part of the majority within the psychologist profession in Sweden. Consequently, this study reflects our understanding of the phenomena of compassion fatigue and it cannot be ruled out that other researchers with other backgrounds would reach other conclusions from the same data.

In conducting this study, we have had the privilege to hear eight practicing psychologists’ own experiences of compassion fatigue, told in an honest and open manner. This honesty inspired us to communicate more openly in our own work as researchers and authors. The two of us who are as of yet inexperienced in working as psychologists have gained invaluable insight in the challenges of our future profession. We have also made sure to maintain clear boundaries between this work and our spare time, so as not to overexert ourselves. It has become clear to us that we need to cultivate sustainable work practices, including a healthy work-life balance, if we are to make it to retirement without burning out.

### Methodological considerations

There is no consensus on which term should be used to describe the cost of caring for others, and various terms are used (Walsh et al., 2017). These include compassion fatigue, secondary traumatic stress, vicarious traumatization, and many others. This study is based on literature found under the recommended key word “compassion fatigue” in PsycINFO, as this encompasses other related terms in the thesaurus. Because of this, it is possible that important literature has been overlooked. To ensure the relevance of the literature, mainly articles concerning psychologists were included, as well as literature pertaining to compassion fatigue in general terms, rather than profession-specific terms.

The process of recruiting participants had certain advantages as well as disadvantages. With a purposive sample, participants who conveyed having first-hand experiences of compassion fatigue were chosen, which increased the relevance of the data. However, the sample might not be representative, since those who suffered the most might be unable to participate, and those who were capable of participating might not have had a representative experience of compassion fatigue. Furthermore, the recruitment was not conducted through randomized sampling, which undoubtedly affected the generalizability of the sample. However, this can be seen as a minor issue in a study that does not claim to draw any causal conclusions from the data.

An additional challenge regarding recruitment was the fact that the participants needed to send in a signed consent form before participating. This led to exclusion of the psychologists who had expressed interest but did not submit their consent forms on time.

A possible improvement in the recruitment process and the research design would have been to let the participants undergo the Compassion Fatigue Self Test (Figley, 2002). This addition would have improved the construct validity of the study.

In order to gather information on compassion fatigue, an interview guide was created. It was based on previous research on compassion fatigue, and provided the participants with several opportunities to share their own thoughts, which was considered a strength of the study. However, the interview guide was undoubtedly skewed, as the researchers’ preconceived ideas influenced which areas were focused on.

Several participants mentioned compassion fatigue as something of a taboo for psychologists. This was not taken into consideration when designing the study, and it cannot be ruled out that the participants omitted certain experiences due to the taboo. However, the participants stated that they had seldom shared their experiences of compassion fatigue with others, and that they saw the interview as an opportunity to ventilate their thoughts and feelings about compassion fatigue.

The participants’ subjective experiences were sometimes intertwined with their psychological expertise during the interviews. This complicated the analysis, as it was difficult to differentiate between their professional or academic knowledge and their own experiences. This expertise is however also one of the greatest strengths of this study, as the participants were able to discuss their own experiences in the light of their expertise, and with proper terms for the psychological phenomena, leaving little room for misunderstandings.

An additional strength of the study is the qualitative research method used, and the thematic analysis that followed. A more inductive analysis, such as grounded theory (Willig, 2013), would have allowed the study to become even more explorative. However, such a method would not allow the focus on organizational factors needed to answer the research questions, and would therefore be a poor fit to the phenomenological aims of this study. In other words, the aims of the study could not have been reached through a more inductive analysis. In order to maintain the semantic epistemological approach, qualitative research methods that claim to interpret the participants’ meanings were ruled out.

### Practical implications

Based on the statements of the participants, this study has various practical implications which can contribute to lower rates of compassion fatigue amongst psychologists. High demands of quantity and logistics were cited by the participants as a straining factor that contributed to them feeling compassion fatigued. This incentivizes organizational changes, such as hiring psychologist assistants in order to alleviate the logistical burden.

Several improvements surrounding leadership were suggested by the participants in this study. One such improvement was regarding the task of prioritizing patient groups. The participants wished for their bosses to make clear decisions on which patient groups should receive care at their clinics, so that the participants’ time and emotional energy could be spent on treatment rather than administration. Within the clear framework resulting from this, the participants wished to be able to make their own decisions regarding which interventions to use, and use their psychological judgement to schedule patients and plan treatments.

Individual strategies considered to be protecting against compassion fatigue include monitoring one’s well-being in order to identify compassion fatigue and other stress related symptoms, in order to make necessary changes in the professional and personal life. Such strategies include setting healthy work-life boundaries, and finding ways to replenish oneself outside of work in order to recuperate.

Lastly, the participants wished for more professional development. This includes supervision at work, and the opportunity of further education, which would help the participants hone their skills, and protect themselves against compassion fatigue.

## Conclusions

This study has shed light on clinical psychologists’ experiences of compassion fatigue. It was shown that the experiences and definitions of the term varied amongst the participants, which highlighted the width of the phenomena.

A prominent factor which was perceived as contributing to compassion fatigue was the logistical burden, which was mentioned by all participants. These tasks were said to take up time that could be used to improve the quality of care, to recuperate, and reflect. This was particularly detrimental when the patient cases were complex and emotionally straining.

Several work-related factors that brought emotional strain were identified, and ethical stress was the most prominent. The participants wished for more influence regarding patient treatment, enabling them to adjust their work procedure to the individual patient. Others wished for their management to take control over the patient wait-list, relieving them of the burden to prioritize. In addition, participants expressed feeling protected by having a supportive boss who had an understanding of the challenges in their daily work, and had reasonable expectations on them. Further research is needed on the effects of leadership style on compassion fatigue.

An unexpected finding was the experience of “Jesus complex” and the perceived expectations of the psychologists’ role. This caused the participants to overexert their ability of feeling compassion, while simultaneously experiencing a taboo surrounding compassion fatigue, hindering them from seeking support. This finding incentivizes the normalization of compassion fatigue to lift the taboo, which in turn will make it easier for suffering professionals to seek help.

All participants stated that they used various methods of monitoring, in order to keep an eye on their psychological and physical well-being. Professional development, such as supervision and education, was cited as a protecting factor against compassion fatigue. Self-care practices, such as going to therapy, and maintaining a healthy lifestyle, were also expressed to be protecting by the participants, and it is recommended to emphasize this aspect in psychologists’ training and education. In addition, more research is needed on different ways to incorporate monitoring in daily work. These recommendations are motivated by the connections between compassion fatigue and burnout, and the societal and individual costs that this connection entails.

## Appendix

**Translated interview guide**

**General information to the participant**

Thank you for participating! Is this a good time for you? My name is X and Y sits next to me. This conversation will be recorded. Your participation is voluntary and anonymous, and you may terminate your participation at any time, without offering an explanation. Nothing you say will be tied to you or your workplace. Have you received and read the information we sent you? Do you have any questions? This interview will take approximately 1–1.5 h of your time, and consists of four different parts; *personal experiences, individual strategies, work-environment*, and *effects of compassion fatigue*.

**Personal experiences**

- Tell me a little bit about your job. What are your work tasks?
- When I say compassion fatigue, what thoughts come to mind?
- Now, I am going to read our definition of compassion fatigue to you: “Compassion fatigue is a condition that commonly occurs in helping professions. The condition is characterized by an gradually eroding ability to experience compassion, an impaired ability to focus, experiences of rumination, a difficulty to practice self-care, and physical stress-related symptoms, such as headaches, nausea and heart palpitations.”
- Have you ever experienced compassion fatigue, and if so, what is your experience of it?
  - If the answer is yes, can you provide me with an example of a typical situation in which you would experience compassion fatigue?
  - If the answer is no, could you imagine a situation where you could experience compassion fatigue?

- Could you provide me with an example of a situation where you could have experienced compassion fatigue, but for some reason, did not?
  - Why do you think you managed to avoid experiencing compassion fatigue in this situation?

**Individual strategies**

- Do you have any strategies to cope with compassion fatigue, and if you do, what are they?
- Do you have any advice to future psychologists on how to manage compassion fatigue?

**Work environment**

- Is compassion fatigue a concept that you discuss/have discussed at your workplace?
  - And if you have discussed it, in what way has the issue been addressed?
- What preconditions at your workplace helps you manage your compassion fatigue?
- What reactions would you have gotten from your co-workers and/or boss if you would have told them that you are suffering from compassion fatigue?
- Have you often experienced difficulty in letting go of thoughts about work when you have your time off?
  - If yes, what is it that makes it difficult to let go of these thoughts?
  - If no, what is it that facilitates you letting go of these thoughts?
- Do you think compassion fatigue is something that often affects psychologists within your domain of work? Why/why not?
- What do you find rewarding about your work?
- What do you find hard/draining about your work?
- How sustainable do you find your current work situation to be?
  - What do you think could make it more sustainable?
  - What would make it less sustainable?

**Effects of compassion fatigue**

- Do you think that compassion fatigue has affected your work situation by any means, and if so, in what way?
- Do you think compassion fatigue has affected your personal life by any means, and if so, in what way?
- Have you ever experienced hopelessness in relation to trying to help your patients, and if so, how has this manifested?
- Do you think that you would have recognized signs of compassion fatigue in a co-worker, and how do you think those signs would manifest?
